# Pancreas-preserving partial duodenectomy for non-ampullary duodenal neoplasms: three case reports

**DOI:** 10.1186/s40792-022-01489-4

**Published:** 2022-07-23

**Authors:** Shunsuke Ishida, Teijiro Hirashita, Yoko Kawano, Hiroki Orimoto, Shota Amano, Masahiro Kawamura, Atsuro Fujinaga, Takahide Kawasaki, Takashi Masuda, Yuichi Endo, Masayuki Ohta, Masafumi Inomata

**Affiliations:** Department of Gastroenterological and Pediatric Surgery, Oita University Faculty of Medicine, 1-1 Hasama-machi, Yufu, Oita 879-5593 Japan

**Keywords:** Duodenal neoplasm, Duodenal adenocarcinoma, Partial duodenectomy, Pancreas-preserving duodenectomy

## Abstract

**Background:**

There are multiple surgical procedures for resecting non-ampullary duodenal neoplasms (NADNs), and the appropriate method is selected depending on the tumor location and diagnosis. We herein report 3 cases of NADNs that were resected using pancreas-preserving partial duodenectomy (PPD).

**Case reports:**

The first patient, a 73-year-old woman with a circumferential duodenal adenoma in the supra-ampullary duodenum, underwent surgery. After laparotomy, the duodenum proximal to the tumor was confirmed using intraoperative endoscopy and dissected. The duodenum distal to the tumor was dissected under direct visualization, and the specimen was removed. The distal stump of the duodenum was closed, and duodenojejunostomy was performed as described by Billroth II. The tumor was diagnosed as an adenoma 75 mm in size. She was discharged 12 days after surgery without any complications. The second patient, a 48-year-old man, was diagnosed with a neuroendocrine neoplasm (NEN) with a diameter of 14 mm in the supra-ampullary duodenum. Laparoscopic PPD was performed. He was diagnosed with NEN G1 and discharged the 11th day after surgery. The third patient, a 71-year-old man with a 0–Is + IIa lesion in the horizontal duodenum, underwent surgery. After laparotomy, the horizontal duodenum and proximal jejunum were resected, and duodenojejunostomy was performed. The patient was diagnosed with stage I adenocarcinoma and discharged on the 15th day after surgery.

**Conclusion:**

PPD is useful for avoiding the morbidity of pancreatoduodenectomy in the management of NADNs without invasion to the ampulla of Vater or pancreas.

## Background

Pancreatoduodenectomy (PD) is often considered a surgical procedure for duodenal tumors. However, PD is a complex surgical procedure with a high rate of postoperative complications. For non-ampullary duodenal neoplasms (NADNs), PD can carry a higher morbidity (including morbidity from pancreatic fistulas) due to the soft pancreatic texture and the small size of the pancreatic duct, which contributes to reduced survival [1, 2]. Pancreas-preserving partial duodenectomy (PPD) is an alternative, less invasive and organ-preserving surgical technique for NADNs that leads to a better postoperative course than PD [3]. Depending on the diagnosis and location of the duodenal tumors, the pancreas may be preserved. We report 3 cases of duodenal tumors that were treated using PPD.

## Case reports

### Patient 1

**Fig. 1:**
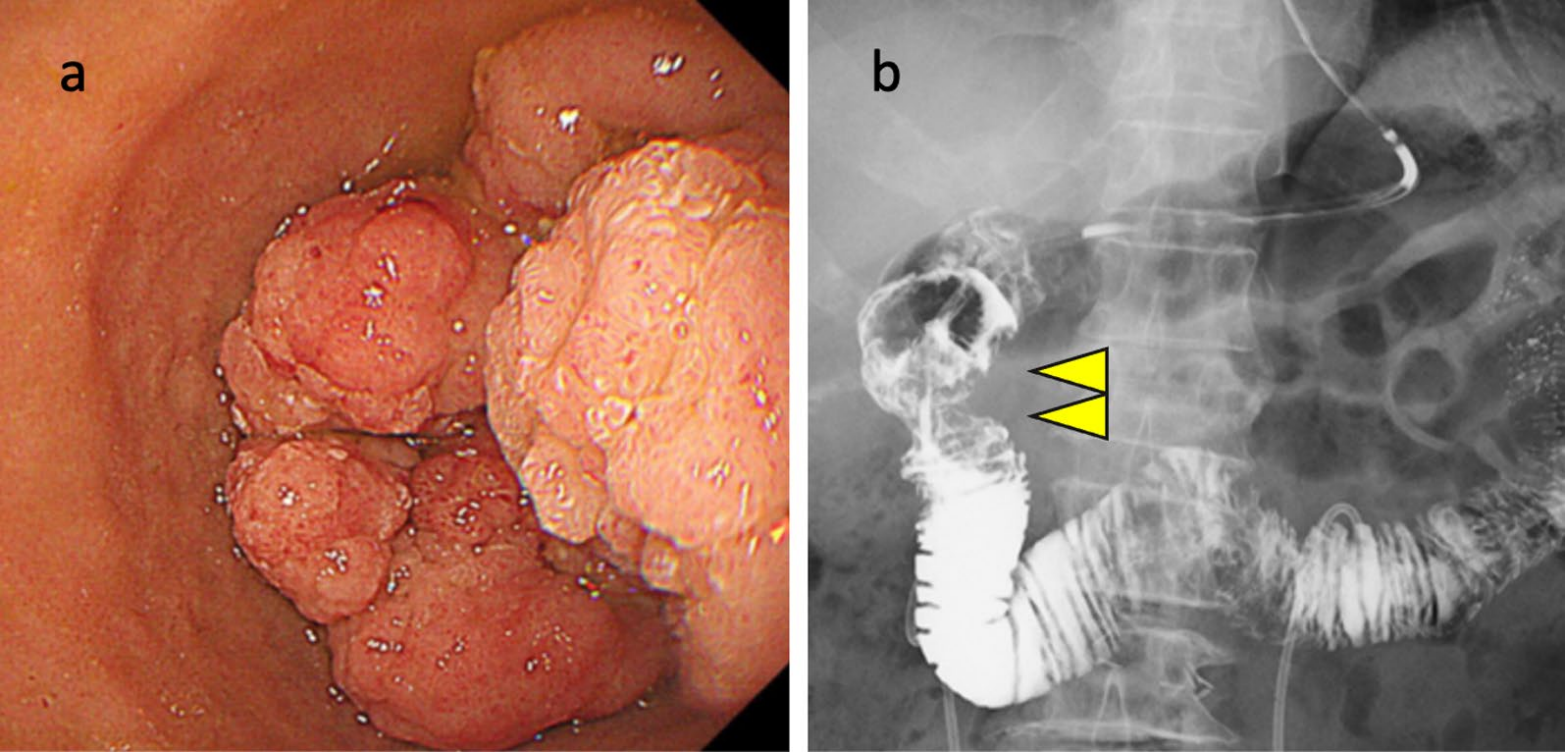
**a** Upper gastrointestinal endoscopy revealed a circumferential papillary tumor located from the duodenal bulb to the descending duodenum. **b** Duodenography revealed a circumferential tumor in the supra-ampullary duodenum (arrowhead)

**Fig. 2:**
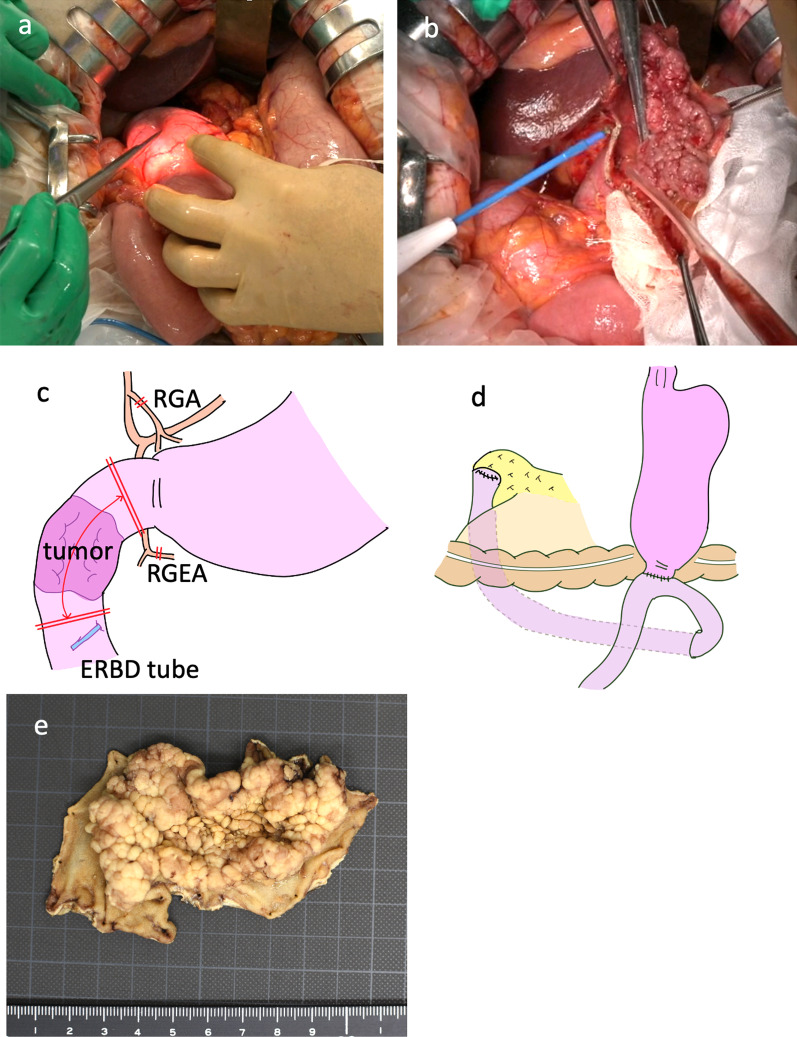
**a** The duodenum proximal to the tumor was confirmed using intraoperative endoscopy. **b** The duodenum distal to the tumor was dissected under direct visualization. **c** Scheme of surgery. *RGA* right gastric artery, *RGEA* right gastroepiploic artery, *ERBD* endoscopic retrograde biliary drainage. **d** Scheme of reconstruction. **e** The specimen showed a papillary tumor 75 mm in size

The first patient was a 73-year-old woman whose duodenal tumor was detected during preoperative examination for ovarian cancer. Upper gastrointestinal endoscopy revealed a circumferential papillary tumor located from the duodenal bulb to the descending duodenum which was diagnosed with duodenal adenoma (Fig. 1a). One year after surgery for ovarian cancer, the duodenal adenoma had grown slightly. Duodenography revealed a circumferential tumor in the supra-ampullary duodenum (Fig. 1b). An endoscopic retrograde biliary drainage (ERBD) tube was inserted preoperatively because the tumor was close to the ampulla of Vater, and pancreas-preserving supra-ampullary duodenectomy was planned. After kocherization, the right gastric and gastroepiploic vessels were dissected. The location of the ampulla of Vater was confirmed using an ERBD tube, and the supra-ampullary duodenum were spared from the pancreas. The duodenum proximal to the tumor was confirmed using intraoperative endoscopy and was dissected. The duodenum distal to the tumor was dissected with preservation of accessory papilla under direct visualization, and the specimen was removed. The distal stump of the duodenum was closed, and duodenojejunostomy was performed as described by Billroth II (Fig. 2). The operation time was 201 min, and blood loss was 100 ml. Pathological examination showed duodenal adenoma. The postoperative course was uneventful, and the patient was discharged on postoperative Day 12.

### Patient 2

**Fig. 3:**
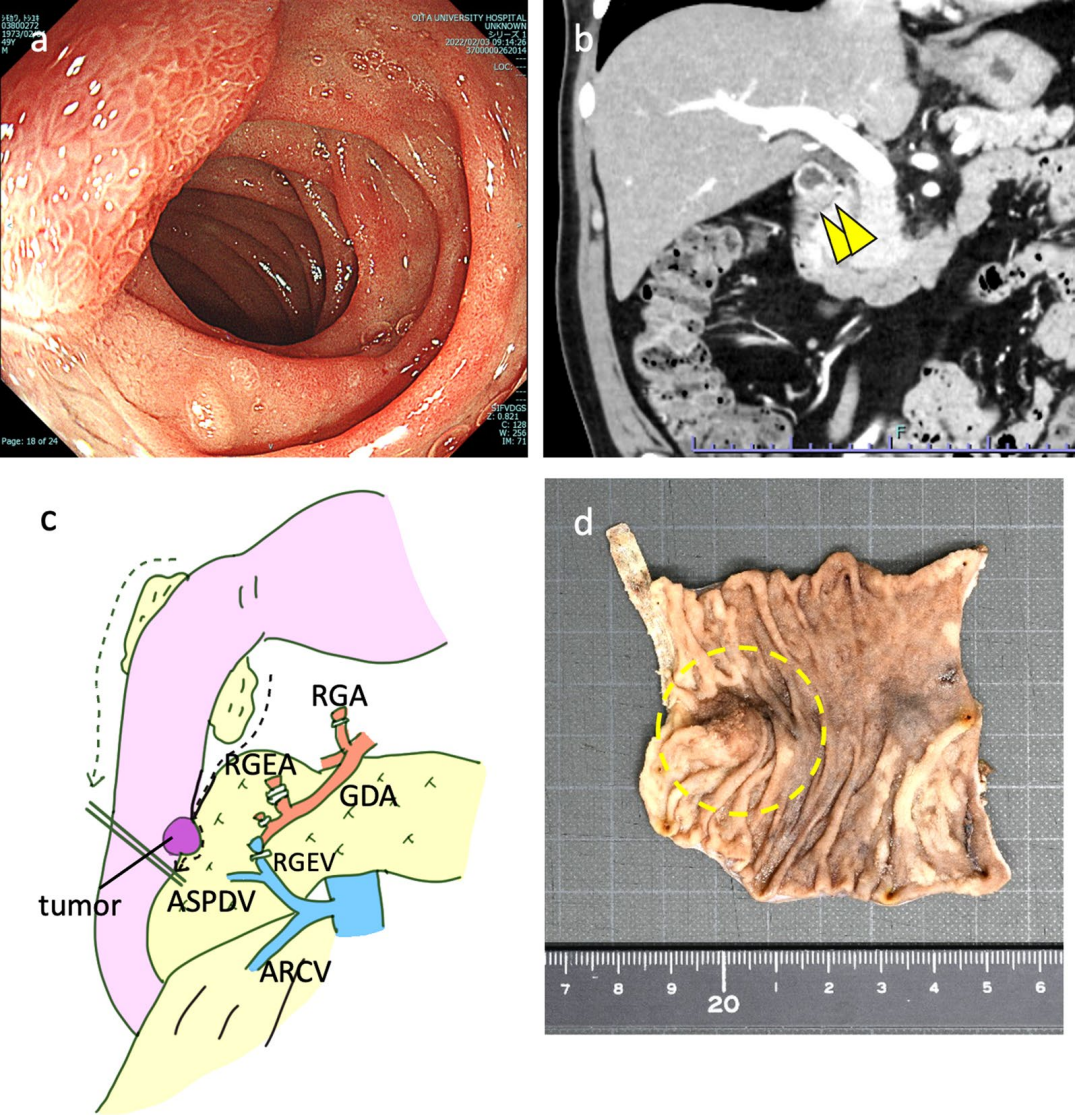
**a** Upper gastrointestinal endoscopy revealed a sessile lesion on the anterior wall of the descending duodenum. **b** CT revealed an 18-mm tumor at the descending part of the duodenum (arrowhead). **c** Scheme of surgery. *RGA* right gastric artery, *RGEA* right gastroepiploic artery, *RGEA* right gastroepiploic vein, *GDA* gastroduodenal artery, *ASPDV* anterior superior pancreatoduodenal vein, *ARCV* accessory right colic vein. **d** The specimen showed a submucosal tumor in the descending duodenal wall

**Fig. 4:**
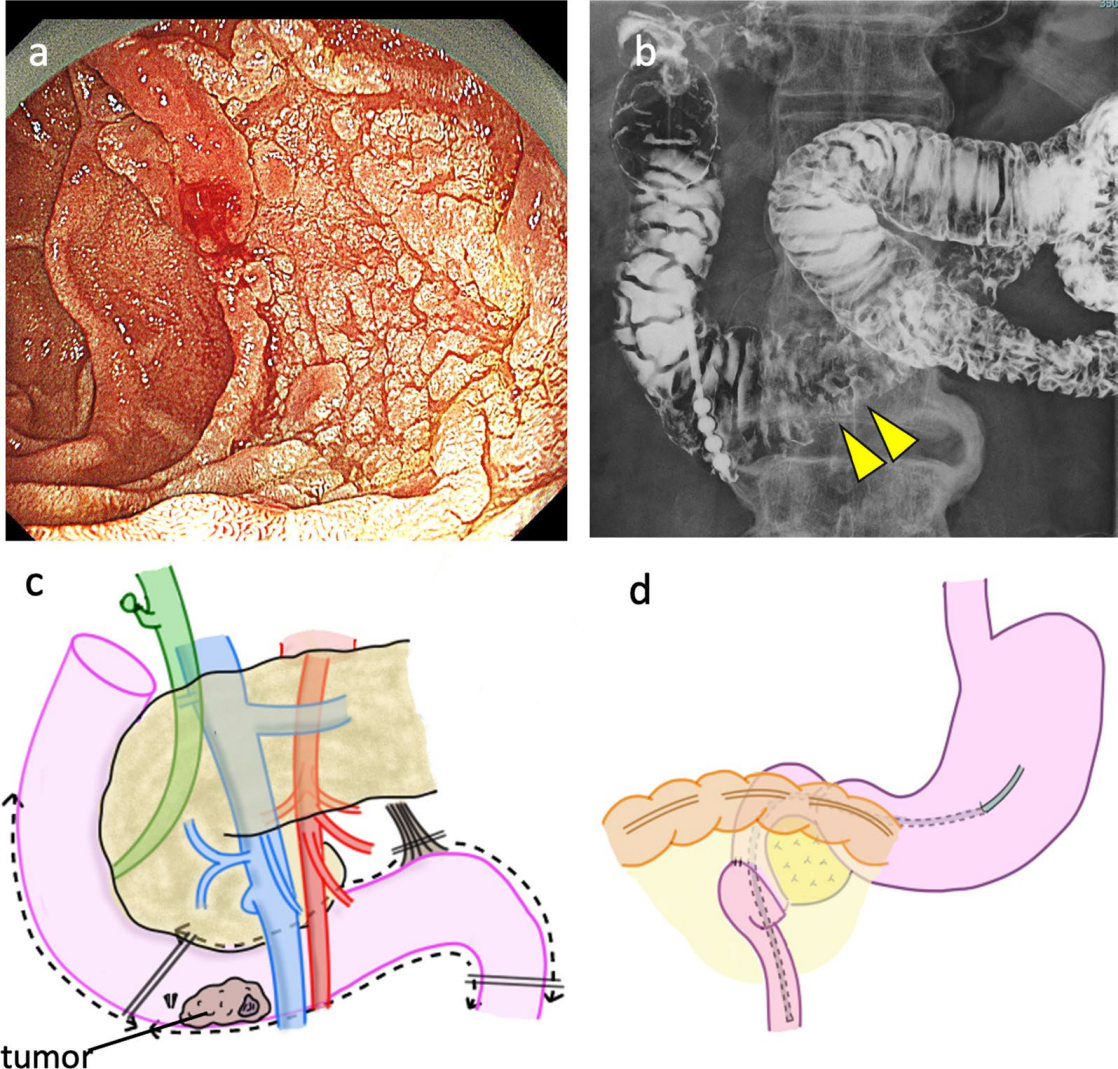
**a** Upper gastrointestinal endoscopy revealed a semi-circumferential 0–Is + IIa lesion in the horizontal duodenum. **b** Duodenography revealed a tumor in the horizontal duodenum. **c**, **d** A diagram of surgical procedures is shown

A 48-year-old man had a flattened elevated lesion 2 cm in size on the anterior wall of the descending duodenum, and a biopsy revealed a neuroendocrine neoplasm (NEN, G1) (Fig. 3a). He underwent left upper lobectomy of the lung for the lung cancer (pT2aN0M0, stage IB) 1 year ago and received adjuvant chemotherapy using tegafur-uracil for 8 months. Endoscopic ultrasonography (EUS) showed that the lesion was approximately 14 mm in diameter and was mainly located in the submucosa. Computed tomography (CT) and duodenography revealed an 18-mm tumor at the descending part of the duodenum (Fig. 3b, c). Laparoscopic pancreas-preserving supra-ampullary duodenectomy was performed. After kocherization, the right gastric and gastroepiploic vessels were dissected and the supra-ampullary duodenum was dissected from the pancreas with No. 5, 6, 13a, and 17a lymph nodes (LNs) dissection. Since only the sparse fibrous tissue was dissected when the duodenum was spared from the pancreas, the accessory papilla would not be damaged. The tumor that protruded from the duodenum was identified, the distal margin was confirmed by intraoperative endoscopy, and the duodenum distal to the tumor was dissected using a linear stapler. The proximal duodenal stump was pulled out from an umbilical incision. The duodenum proximal to the tumor was dissected under direct visualization, and the specimen was removed. Duodenojejunostomy was performed as described by Billroth II (Fig. 4). The operation time was 306 min, and blood loss was 20 ml. Pathological examination showed duodenal NEN, G1. The postoperative course was uneventful, and the patient was discharged on postoperative Day 11.

### Patient 3

A 71-year-old man had a semi-circumferential 0–Is + IIa lesion in the horizontal duodenum (Fig. 4a, b), and it was diagnosed as a duodenal cancer by biopsy. He had undergone cholecystectomy for gangrenous cholecystitis 10 years ago. After endoscopic clips were placed at the duodenum just proximal to the tumor, pancreas-preserving infra-ampullary duodenectomy was performed. After laparotomy, the proximal jejunum was dissected from the pancreas. The duodenum at the inferior duodenal angulus was dissected proximal to the clips. Only the LNs around infra-ampullary duodenum were resected because of the preoperative diagnosis of T1a. Duodenojejunostomy was performed using functional end-to-end anastomosis (Fig. 4c, d). The operation time was 307 min, and blood loss was 347 ml. Pathological examination showed duodenal adenocarcinoma, 28 mm in diameter, and pathological stage T1aN0M0 Stage I (TNM classification). The postoperative course was uneventful, and the patient was discharged on postoperative Day 15.

## Discussion

The treatment strategies for NADAs are unclear. Tumors that are not amenable to endoscopic resection require operative intervention. PD has been the standard operation for invasive lesions that involve the ampulla of Vater or that require lymph node dissection. PPD are usually indicated for select tumors, including gastrointestinal stromal tumors, large adenomatous lesions, T1a adenocarcinoma, and small NEN, that has no risk of LN metastasis. The choice of PPD versus PD is dependent on factors, such as tumor size, location (proximity to the ampulla of Vater), risk of lymph node metastasis, and patient’s overall fitness. However, there are few reports comparing oncological results between PD and PPD, and an ideal surgical approach has not yet been established. Including total duodenectomy, supra- or infra-ampullary partial duodenectomy, and wedge resection of the duodenum, PPD may be one of the curative treatments for non-ampullary duodenal neoplasms depending on the tumor location and diagnosis [3]. When PPD is performed, handling of the accessory papilla is important. Since its damage leads to postoperative complication including pancreatic fistula, it should be preserved if possible. If a communication between the Santorini and the Wirsung duct is confirmed, it can be sutured or ligated. It is necessary to confirm it by MRCP or ERCP before surgery when the accessory papilla is planned to be ligated or sutured.

PPD has several advantages over PD in terms of operative complexity and organ preservation that leads to a lower incidence of postoperative complications and preservation of pancreatic function. There are some reports that show the usefulness of PPD, but the overall survival was not significantly different, although patients undergoing PD had a higher morbidity [4–6]. An important limitation of PPD is insufficient oncological resection due to the absence of LN dissection. In early non-ampullary duodenal adenocarcinoma (NADA), many reports showed that there was no incidence of LN metastasis in the NADA limited to the mucosa [7]. Otherwise, in reports that examined small numbers, high rates of LN metastasis in submucosal invasive NADA were reported, ranging from 14 to 42% [8–10]. In addition, there is no crucial opinion on the range of LN dissection depending on the tumor location; furthermore, the optimal range of LN dissection for the NADA in the horizontal and ascending parts of the duodenum is unknown. In a case of NADA located in the horizontal or ascending duodenum PPD may be sufficient even in advanced stages because LN dissection around the superior mesenteric artery could be performed [11, 12]. It is better to avoid unnecessary PD if the tumor does not invade the pancreas directly, especially early NADA. Although patients 1 and 3 were diagnosed with adenoma and T1a adenocarcinoma, respectively, endoscopic treatment or local resection was difficult to perform without complications due to tumor size and location. EUS could be useful for more accurate preoperative diagnosis in terms of depth of invasion, though it has not been performed on these patients. We performed PPD in both cases and negative margins were achieved. There was no risk of LN metastasis; therefore, the selected procedures were better than PD.

We performed partial duodenectomy rather than local resection because of lymph node dissection around the supra-ampullary duodenum for the second patient with NEN. Soga et al. [5] reported that the LN metastasis rate of duodenal NENs among 655 patients was 10.6% for tumor diameters of 5 mm or less, 13.9% for 6–10 mm, 24.7% for 1.1–2.0 cm, and 24.7% for tumors above 2.0 cm. In a retrospective study conducted in Japan, risk factors for metastasis were reported to be NET G2, multiple tumors, tumor size greater than 1.1 cm, and positive vascular invasion [6]. Although PD is superior to PPD in terms of lymph node dissection, PPD can be selected for a relatively small NEN if negative margins can be confirmed and there is no LN swelling on the preoperative images. Since the second patient did not undergo PD, it is necessary to follow up strictly for recurrence, including checking LNs.

The anatomical features of the duodenum, such as the narrow lumen and thin wall, make endoscopic resection of tumors difficult. To prevent complications of ESD, laparoscopic–endoscopic cooperative surgery (LECS), in which the defect of the duodenal wall by endoscopic procedure is closed by laparoscopic suture from the outside of the duodenum, was recently implemented to treat patients with NADA [13]. Although LECS requires complicated procedures and surgical instruments, it is an effective backup technique [14]. Duodenal neoplasms located at the opposite side of the ampulla of Vater, which are difficult to resect by partial duodenectomy, can be resected by the LECS that can determine the excision range and repair the defect of duodenal wall after excision. Although we did not perform LECS for our 3 patients because of tumor size and location, LECS would be useful for a certain number of cases with NADA.

We agree that PPD is an attractive and promising alternative procedure to PD for the treatment of NADNs, especially small NENs, gastrointestinal tumors, large adenomas, and early NADAs. The technical points and pitfalls of this operation have not been sufficiently discussed thus far. Further large studies to examine the safety and curability of PPD are needed in the future.

## Conclusions

PPD is useful in the management of non-ampullary duodenal neoplasms without invasion to the ampulla of Vater or pancreas, avoiding the morbidity of PD. Appropriate preoperative examinations and treatment strategies should be conducted with consideration for organ preservation and curability.

## Data Availability

The data are not available for public access because of patient privacy concerns but are available from the corresponding author on reasonable request. All authors read and approved the final manuscript.

## Abbreviations

NADN: Non-ampullary duodenal neoplasm
PD: Pancreatoduodenectomy
PPD: Pancreas-preserving duodenectomy
ERC: Endoscopic retrograde cholangiography
ERBD: Endoscopic retrograde biliary drainage
NEN: Neuroendocrine neoplasm
EUS: Endoscopic ultrasonography
CT: Computed tomography
LN: Lymph node
NADA: Non-ampullary duodenal adenocarcinoma
